# Revolutionizing cardiovascular medicine: targeted therapies for the cardiac conduction system

**DOI:** 10.1172/JCI164192

**Published:** 2022-10-17

**Authors:** Daniel J. Garry, Demetris Yannopoulos, Tamas Alexy

**Affiliations:** 1 Cardiovascular Division, Medicine Department,; 2 Regenerative Medicine and Sciences Program,; 3 Stem Cell Institute, and; 4 Center for Resuscitation Medicine, University of Minnesota, Minneapolis, Minnesota, USA.

## Abstract

Arrhythmogenic cardiovascular disorders are associated with considerable morbidity and mortality. Whether cardiac conduction disease is caused by genetic defects, procedural perturbations, valvular disease, ischemia, aging, or heart failure, new therapies are warranted. In this issue of the *JCI*, Goodyer et al. used state-of-the-art technologies to image the cardiac conduction system (CCS) in real time and to deliver targeted therapies to the CCS and its subcomponents. These findings advance the ability to image and treat specific lineages within the adult heart with the potential for broader applications in the treatment of cardiovascular diseases.

## The impact of arrhythmias and cardiovascular health

Cardiovascular disease is the number one cause of death in the United States and worldwide (1). In the United States alone, nearly 400,000 individuals per year have an out-of-hospital cardiac arrest. Primary arrhythmic events are the cause in a substantial proportion of those patients (2). Nonfatal arrhythmias are even more frequent, as atrial fibrillation affects more than 3 million Americans. Given the anatomical location of the cardiac conduction system (CCS), postcardiotomy patients are also at risk for arrhythmias due to structural perturbations of the myocardium and the CCS (3). The development of targeted therapies and the prevention of surgical complications would be accelerated with the capability of imaging the CCS in real time. In addition, the ability to target the entire CCS (or a subpopulation of cells that comprise an anatomically distinct conduction segment) provides therapeutic opportunities with decreased side effects or off-target effects of bystander cell populations.

## Live visualization of the CCS

**Figure 1:**
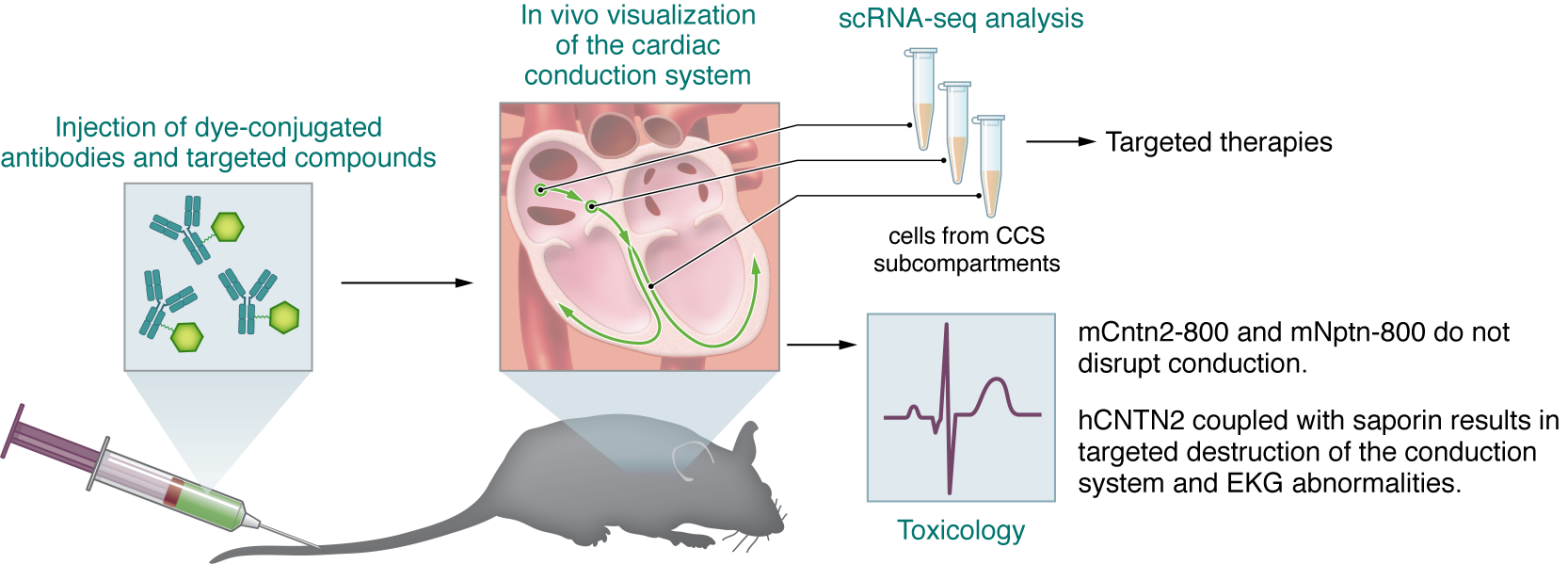
Imaging and targeting the CCS in the adult heart. Goodyer and colleagues generated dye-conjugated antibodies against cardiac conduction–restricted proteins. mCntn2-800 (or mNptn-800) injected intravenously into mice provided a robust signal in adult heart, allowing for live visualization of the CCS. Engineered antibodies that recognize CCS-specific proteins could also deliver targeted compounds to modulate conduction. An anti–human CNTN2 antibody coupled with the cellular toxin saporin (hCNTN2-Sap) resulted in conduction abnormalities, while mCntn2-800 and mNptn-800 did not disrupt the CCS, as measured by ECG. Isolation of single cells from different CCS regions followed by scRNA-seq revealed cell surface markers that might be used to visualize specific parts of the CCS or conjugated with therapeutic cargo to treat cell-specific conduction anomalies (4).

In this issue of the *JCI*, Goodyer et al. (4) describe a strategy that has high resolution and specificity to image the CCS that could be used for diagnostic purposes, electrophysiological treatments, intracardiac surgical reconstruction, or valvular surgical procedures. To demonstrate the feasibility of the optical imaging strategy, Goodyer et al. conjugated a commercially available near-infrared (NIR) dye to a polyclonal antibody directed against the CCS-restricted cell surface marker, contactin 2 (CNTN2). This antibody-dye conjugate (mCntn2-800), targeted against the murine Cntn2 protein, was injected as a single dose intravenously into adult mice, followed by organ removal and analysis using closed-field NIR imaging 72 hours later. mCntn2-800 signal was detected in kidneys and liver (as anticipated), and showed a high-intensity signal localized to the CCS (compared with the IgG control) over a range of doses. A single dose of 75 μg had a robust signal for up to four days from the initial injection. The specificity of the antibody-dye conjugate was further verified using immunohistochemical techniques with other known CCS markers and tissue clearing of the entire heart (following the delivery of 75 μg of mCntn2-800) demonstrated signal throughout the entire CCS (Figure 1). Using a FLARE intraoperative NIR fluorescence imaging system (already used in clinical trials) (4), the researchers performed a sternotomy and imaged the NIR signal in real time, demonstrating the feasibility of imaging the CCS during clinical procedures (4). To further translate these findings, a monoclonal antibody was generated against human CNTN2 and was conjugated to the same dye (hCNTN2-800) and shown to bind and detect the CCS following tail vein injection in adult mice (Figure 1).

The ability to target the CCS with high degree of sensitivity and specificity then allowed Goodyer et al. (4) to pursue further studies focused on high precision delivery of drugs, modifying agents, or other cargo to the CCS. The investigators used the monoclonal antibody against hCNTN2 (biotinylated) and conjugated to streptavidin that was bound to saporin (Sap), which is a cellular toxin. The agent was then delivered as a single dose intravenously to adult mice (Figure 1). Daily electrocardiograms (ECGs) dem0onstrated the onset of conduction abnormalities (prolonged PR and QRS intervals) and cell death involving the CCS in the hCNTN2-Sap experimental group compared with controls (4). These studies further support the feasibility of specifically targeting the CCS with compounds of interest, ablation reagents, and antiarrhythmics to treat disorders of the CCS.

Single-cell RNA sequencing (scRNA-seq) has enhanced our understanding of the complexity of heart development, growth, and disease (5–12). An array of publicly available databases such as the Human Cell Atlas now allows investigators to examine the molecular profile of anatomically distinct cell populations using the single-cell data sets (9, 12). Goodyer and colleagues used computational analysis of their single-cell data sets and screened subpopulations (sinoatrial node, atrioventricular node, Purkinje fibers, etc.) of the CCS to discover cell surface proteins that were enriched in the CCS (4). These mining initiatives revealed several candidates, including neuroplastin (Nptn), which is a transmembrane protein expressed in the adult heart and brain in mouse and human (4, 13). Goodyer et al. demonstrated that Nptn was expressed throughout the entire CCS in the adult human and mouse. Using a previously described strategy, the intravenous delivery of mNptn-800 and the use of live imaging demonstrated that the restricted, specific signal was localized to the CCS (4).

## Conclusions and future directions

There are a number of exciting discoveries that emerge from the studies of Goodyer et al. (4) and they will serve as a platform for clinical applications and further studies. Cellular and molecular imaging modalities described here will provide an unprecedented three-dimensional outline of the CCS, which will advance our understanding of arrhythmic perturbations associated with conduction defects, congenital heart defects, a variety of diseases, and aging. Extension of these studies using large animal models, such as the domestic pig, will outline whether these applications are ready for the clinic or whether further refinements will be necessary. While no conduction perturbations were observed with the single dose of the mCntn2-800 or mNptn-800 antibodies, longer surveillance studies will be needed to be performed. Additionally, it is unclear whether these antibody-based imaging methods can be repeatedly used and over what time period can they be administered.

Another discovery that emerged from Goodyer et al. (4) was the ability to target the CCS or subcomponents of the CCS for the delivery of a therapeutic agent. The foundation for these discoveries emerged from the analysis of scRNA-seq data sets. These single-cell data sets emphasize the complexity of the CCS and other cardiac subcomponents but they also serve as an important mining tool for therapeutics (8–10, 14). The molecular ablation strategies will need to be further examined in large animal models as well as the degree (or completion) of the ablation method as a prelude to clinical studies.

In summary, the studies by Goodyer et al. emphasize the importance and the accelerated pace of moving from bench to bedside to address important clinical problems (4). They further mark a paradigm shift from the treatment of organs and tissues to the targeting of single cells or subcomponents such as the CCS in the adult heart. These studies signify an exciting chapter in cardiovascular medicine focused on single-cell (or lineage specific) therapeutics.

## Version 1 10/17/2022

Electronic publication

## References

[1] Heidenreich PA (2022). Economic issues in heart failure in the United States. J Card Fail.

[2] Link MS (2015). Part 7: Adult Advanced Cardiovascular Life Support: 2015 American Heart Association guidelines update for cardiopulmonary resuscitation and emergency cardiovascular care. Circulation.

[3] Kerola T (2019). Risk factors associated with atrioventricular block. JAMA Netw Open.

[4] Goodyer W (2022). et al. In vivo visualization and molecular targeting of the cardiac conduction system. J Clin Invest.

[5] Nakada Y (2022). Single nucleus transcriptomics: apical resection in newborn pigs extends the time window of cardiomyocyte proliferation and myocardial regeneration. Circulation.

[6] Gong W (2022). ETV2 functions as a pioneer factor to regulate and reprogram the endothelial lineage. Nat Cell Biol.

[7] Gong W (2017). Dpath software reveals hierarchical haematoendothelial lineages of Etv2 progenitors based on single-cell transcriptome analysis. Nat Commun.

[8] Litvinukova M (2020). Cells of the adult human heart. Nature.

[9] DeLaughter DM (2016). Single-cell resolution of temporal gene expression during heart development. Dev Cell.

[10] Goodyer WR (2019). Transcriptomic profiling of the developing cardiac conduction system at single-cell resolution. Circ Res.

[11] Li G (2019). Single cell expression analysis reveals anatomical and cell cycle-dependent transcriptional shifts during heart development. Development.

[12] Asp M (2019). A spatiotemporal organ-wide gene expression and cell atlas of the developing human heart. Cell.

[13] Herrera-Molina R (2017). Neuroplastin deletion in glutamatergic neurons impairs selective brain functions and calcium regulation: implication for cognitive deterioration. Sci Rep.

[14] Reichart D (2022). Pathogenic variants damage cell composition and single cell transcription in cardiomyopathies. Science.

